# *LOXL1* expression in lens capsule tissue specimens from individuals with pseudoexfoliation syndrome and glaucoma

**Published:** 2010-11-02

**Authors:** Tanya T. Khan, Guorong Li, Iris D. Navarro, Rama D. Kastury, Carol J. Zeil, Taras M. Semchyshyn, Frank J. Moya, David L. Epstein, Pedro Gonzalez, Pratap Challa

**Affiliations:** Duke University Eye Center, Durham, NC

## Abstract

**Purpose:**

To study lysyl oxidase-like 1 (*LOXL1*) expression in freshly collected lens capsules from pseudoexfoliation syndrome (XFS), pseudoexfoliation glaucoma (XFG), and normal cataract control individuals. We also investigated the effects of four glaucoma drug medications on *LOXL1* expression in primary human lens epithelial cell cultures to see if they could affect *LOXL1* expression.

**Methods:**

Lens capsules were collected at the time of cataract surgery. Controls were matched to age, sex, and ethnicity. Total RNA was isolated from individual lens capsule samples and real-time PCR was performed on each sample using primers flanking the sixth exon of the *LOXL1* gene. Cell cultures were grown to confluence in four separate six-well plates at 37 °C in 5% CO_2_. Each plate was then treated with one of four different glaucoma drugs (brinzolamide 1%, brimonidine tartrate 0.1%, timolol maleate 0.5%, and latanoprost 0.005%) once daily for seven days (at both 1:1,000 and 1:100 concentrations relative to media). Controls were not treated with any drug but media was changed in the same manner. After one week of treatment, cells were harvested and total RNA isolated. Real-time PCR was performed on each group of cells.

**Results:**

Seven XFS, seven XFG, and ten cataract control specimens were analyzed. *LOXL1* expression was detected in the lens capsule specimens from each of the four groups. Significant expression differences were found between the control and XFG groups and XFS and XFG groups. No significant difference was observed between the control and XFS group. No significant decrease in *LOXL1* expression was seen with drug incubation of the four medications (Brinzolamide, Timolol, Latanoprost, and Brimonidine) at the 1:1,000 drug:media concentrations versus controls. At 10-fold higher concentrations (1:100 drug:media), brinzolamide, timolol maleate, and latanoprost showed small increases in *LOXL1* expression relative to controls. This effect was not observed with brimonidine tartrate.

**Conclusions:**

These results establish that *LOXL1* expression is reduced in lens capsule specimens from XFG individuals but not XFS. The drug treatment incubation studies suggest that the change in *LOXL1* expression observed in XFG is not attributable to glaucoma drug therapy. If a causative functional relationship can be validated, modification of *LOXL1* expression in affected tissues may represent a novel treatment strategy for this disorder.

## Introduction

Pseudoexfoliation syndrome (XFS) is an age-related systemic disorder of the extracellular matrix that is characterized by abnormal microfibrillar production and deposition in intra- and extraocular tissues [1]. Structures in the anterior segment of the eye are most often involved, including the lens, the iris, the ciliary body, and the zonular apparatus [2]. Progressive pathological accumulation of pseudoexfoliation deposits both from local production and secondary deposition from the aqueous humor appears to lead to obstruction of the trabecular meshwork and subsequent elevation in intraocular pressure [3]. This can lead to optic nerve damage and the development of pseudoexfoliation glaucoma (XFG) which is the most common identifiable cause of secondary open-angle glaucoma in the world [4]. Compared to primary open-angle glaucoma (POAG), patients with XFG demonstrate more rapid pressure increase, resistance to medical therapy, and more need for glaucoma surgery [5].

Disease prevalence is estimated at 10% to 20% of the general population over the age of 60 years [6], and increases to 40% in individuals 80 years and older [7]. XFS is observed worldwide with some evidence of geographical clustering. High prevalence rates have been reported in the Navajo Indian population (38%) [8], followed by Scandinavia (i.e., Iceland and Finland) at approximately 20%–25% [9]. In northern Sweden, Astrom et al. [10] concluded that XFS affects every fourth individual approaching 66 years of age. In contrast, the Framingham Eye Study from the United States established a step-wise, age-correlated incidence in non-glaucoma individuals that increases from a much lower 0.6% at 52–64 years of age to 5.0% at 75–85 years of age [11]. By far, the lowest incidence of XFS is found in Eskimo populations in which it is almost non-existent [9].

In a recent genomic association study, Thorleifsson et al. [12] identified three single nucleotide polymorphisms (SNPs) of the lysyl oxidase-like 1 (*LOXL1*) gene as important genetic susceptibility factors for XFS and XFG in Icelandic and Swedish populations. Subsequent replication studies performed in the United States [13-17], Australia [18], and Europe [19], have confirmed that two nonsynonymous coding SNPs (rs3825942 and rs1048661) and one intronic SNP (rs2165241) from *LOXL1* are genetic susceptibility factors for XFS and XFG. In Indian [20], Japanese [21-26], and Chinese [27,28] cohorts, the association with rs3825942 was also replicated and confirmed that this is the strongest risk allele across different ethnicities. However, the causative nature of these SNPs is unclear since other studies have shown inverse relationships for the reported risk alleles of rs3825942 (G), rs1048661 (G), and rs2165241 (T). The rs3825942 has an inverse relationship among individuals from South Africa [29] while rs1048661 and rs2165241 are inversely related among Japanese [21-26] and Chinese [28] cohorts. Therefore, the functional significance of the *LOXL1* gene in the pathogenesis of XFS and XFG is unclear at present.

*LOXL1* is located on chromosome 15q24.1 and is part of a family of five lysyl oxidase enzymes (LOX, LOXL1, LOXL2, LOXL3, and LOXL4) that collectively play a key role in cross-linking between collagen and elastin in connective tissues [30]. Individually, LOXL1 catalyzes tropoelastin cross-linking and regulates elastin fiber formation and remodeling [31]. Moreover, a growing body of molecular and biochemical evidence indicates that XFS arises from a stress-induced elastic microfibrillopathy. Although the exact pathogenesis of XFS remains unknown, it is believed to involve inadequate breakdown and/or excessive production of elastic fiber components [32,33]. Preliminary studies from cadaveric pseudoexfoliation ocular tissues reveal a reduction of *LOXL1* gene expression in both advanced XFS (20% reduction) and XFG (40% reduction) but not early XFS [34]. Furthermore, LOXL1 was identified as a major component of the abnormal fibrillar material accumulated in XFS and XFG. Based on these findings, we choose to study *LOXL1* expression patterns in XFS and XFG using freshly collected lens capsules from XFS, XFG, and normal control individuals undergoing cataract surgery. We also studied the effect of four glaucoma drug medications on *LOXL1* expression in primary human lens epithelial cell cultures to see if they affect *LOXL1* expression.

## Methods

### Lens capsule collection

Duke University IRB approval was obtained before starting the study and informed consent was obtained from all participating individuals. All patients were examined by board certified glaucoma or cornea specialists. Lens capsules were collected at the time of cataract surgery, immediately stored in RNAlater® (Ambion Inc., Austin, TX), and subsequently stored at −80 °C until analysis. Controls were matched to age, sex, ethnicity, and type of cataract.

Pseudoexfoliation changes were identified as the presence of a central disk of XFS material, a clear annular zone (partial or complete), or flakes of XFS material on the lens surface, iris, or corneal endothelium in either eye. Patients were excluded if there was a history of exposure to intense infrared light e.g., glassblowing is associated with true exfoliation of the lens capsule rather than XFS. XFG was diagnosed when patients possessed the above XFS characteristics and at least two of the following criteria: A) documented intraocular pressure (IOP) ≥22 mmHg in either eye; B) glaucomatous optic nerve cupping defined as a cup to disc ratio >0.7 in either eye, notching of the neuroretinal rim, or an asymmetric cup to disc ratio >0.2; and/or C) glaucomatous visual field loss consistent with the optic nerve appearance. Glaucoma suspects were excluded from this study. Controls were individuals of similar age as the patients without any evidence of glaucoma or pseudoexfoliation deposits on intraocular tissues. Their IOPs were in the normal range (<21 mmHg) with normal-appearing optic nerves.

### Cell cultures and medication treatment

Post-mortem human eyes were obtained within 2 days post-mortem according to the tenants of the Declaration of Helsinki. Eyes were obtained from a 42-year-old Caucasian male, a 50-year-old Asian male, and a 50-year-old Hispanic female. Briefly, extracted lens capsules (free of any adherent tissues) were cut into small pieces and digested in medium 199 containing 1.5 mg/ml collagenase IV and 0.2 mg/ml porcine albumin at 37 °C for 60 min. At the end of the digestion, the contents were centrifuged 100× g for 10 min at 22 °C, and the cell pellet was suspended in Dulbecco's modified Eagle medium (DMEM) containing 20% fetal bovine serum (FBS), penicillin (100 Units/ml), streptomycin (100 μg/ml), and gentamicin (20 μg/ml), then plated on plastic Petri plates coated with 2% gelatin. Cell cultures were grown at 37 °C, and under 5% C0_2_. Second to fourth passages were used throughout this study. All reagents were obtained from Invitrogen Corporation (Carlsbad, CA).

Cell cultures were grown to confluence in four separate six-well plates (Costar 3516; Costar, Cambridge, MA). Each plate was then treated with one of four different glaucoma drugs once daily for seven days (at 1:1,000 or 1:100 concentrations relative to media) with simultaneous change of media. The 1:1,000 concentration was chosen based on the relative bioavailability of topical medications given the barriers to ocular drug permeation such as the precorneal tear film, corneal epithelial barrier, dilution in the aqueous humor, and preferential flow toward the trabecular meshwork (and hence away from the lens) [35]. Since ocular bioavailability is highly variable, a second 1:100 concentration was studied to represent a 10-fold increase in drug concentration. These drugs were chosen because they were used by the XFG individuals studied and they represent four separate classes of glaucoma medications with distinct pharmacology. They included: brinzolamide 1%, brimonidine tartrate 0.1%, timolol maleate 0.5%, and latanoprost 0.005%. Controls were not treated with any drug but media was changed in the same manner. After one week of treatment, cells were harvested.

### RNA isolation and quantification

Total RNA was isolated from individual lens capsule samples using an RNeasy kit (Qiagen Inc., Valencia, CA) according to the manufacturer’s instructions and then treated with DNase. RNA yields were measured using RiboGreen fluorescent dye (Molecular Probes, Eugene, OR).

### Real-Time PCR

First strand cDNA was synthesized from 0.5 μg of total RNA by reverse transcription using oligodT and Superscript II reverse transcriptase (Invitrogen Corporation, Carlsbad, CA) according to the manufacturer’s instructions. Quantitative polymerase chain reactions (Q-PCR) were performed in a 20 µl mixture that contained 1 µl of the cDNA preparation and 1× iQ SYBR Green Supermix (Bio-Rad, Hercules, CA), 3 mM MgCl_2_, and 1 mM of primer. PCR parameters were as follows: 95 °C for 5 min followed by 50 cycles of 95 °C for 15 s, 65 °C for 15 s, and 72 °C for 15 s. The fluorescence threshold value (C_t_) was calculated using the iCycle system software (Bio-Rad). The absence of nonspecific products was confirmed by analysis of the melt curves. Primers flanking the sixth exon of *LOXL1* were selected and optimized. β-Actin (*ACTB*) was used as an internal standard of mRNA expression and to normalize gene expression levels. The primers and conditions used for Q-PCR amplification are shown in Table 1.

**Table 1:** Primers used for Q-PCR amplification of a 220 bp fragment of *LOXL1* (accession number: NM_005576.2).

**Gene**	**Sequence**	**Primer E value**
*LOXL1* F	5′-AGCGCTATGCATGCACCTCTCATA-3′	E: 10−4
*LOXL1* R	5′-TGCAGAAACGTAGCGACCTGTGTA-3′	E: 10−4
*ACTB* F	5′-CCTCGCCTTTGCCGATCCG-3′	
*ACTB *R	5′-GCCGGAGCCGTTGTCGACG-3′	

*ACTB* (accession number: NM_001101.3) control sequences are also as listed. F=forward primer and R=reverse primer. E=Primer expected value.

## Results

### Lens capsule collection

All participants underwent uncomplicated clear-corneal phacoemulsification cataract surgery for nuclear sclerotic cataracts. A total of 28 samples were analyzed: seven XFS, seven XFG, ten cataract control specimens, and four for quality-control. Individual specimen yields were too low to perform both quality analysis and Q-PCR; therefore, 4 representative samples were randomly selected for the quality-control analysis. The ages for each group were not statistically significantly different and the averages were as follows: XFS (76.3), XFG (73.5), control (76.5), and quality-control (73.4). Each capsule specimen was approximately 5 mm in diameter. RNA yields were determined using the Ribogreen fluorescent dye (Molecular Probes Inc., Eugene, OR) and RNA quality was confirmed using the Agilent 2100 Bioanalyzer (Agilent Technologies Inc., Santa Clara, CA).

### Expression of *LOXL1* in lens capsule specimens

**Figure 1:**
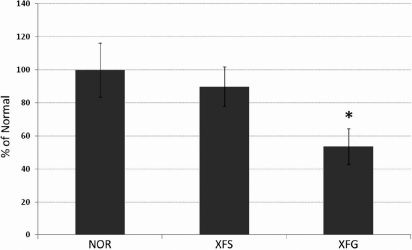
Tabulated *LOXL1* gene expression in lens capsule specimens expressed as a percentage of measured expression in normal specimens. NOR=normal cataract, XFS=pseudoexfoliation syndrome, XFG=pseudoexfoliation glaucoma, and * denotes a p-value of <0.02. A significant difference in expression was observed between the NOR and XFG and XFS and XFG comparisons. No significant difference was seen between NOR and XFS.

A total of 24 samples were analyzed: seven XFS, seven XFG, and ten cataract control specimens. *LOXL1* mRNA expression was detected in all lens capsule specimens analyzed from the three different groups (Figure 1). Gene expression was measured and normalized to control tissue expression. Expression levels were similar in control and XFS lens tissue, whereas decreased expression was measured in XFG specimens (53% of control levels [p<0.02]). P-values using Student’s *t*-tests demonstrated significant differences between both the control and XFG and XFS and XFG groups. No significant difference was observed between the control and XFS group.

### *LOXL1* expression in treated hLEC

**Figure 2:**
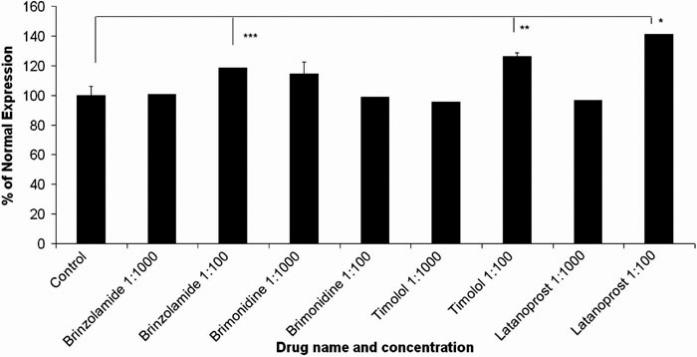
*LOXL1* gene expression in drug-treated (1:1,000 and 1:100) hLEC using real-time PCR. Data were normalized to β-actin (*ACTB*). Values represent percent of normal expression in controls ±SEM of three separate experiments. No significant decrease in expression was observed in any of the samples. Significant increases in expression were seen in the Brinzolamide 1:100, Timolol 1:100, and Latanoprost 1:100 groups. * denotes a p-value <0.003, ** denotes a p-value <0.03, and *** denotes a p-value <0.05.

Real-time semi-quantitative PCR revealed no significant difference in *LOXL1* expression between the control groups and the 1:1,000 drug:media groups incubated with brinzolamide, brimonidine, timolol maleate, or latanoprost (see Figure 2). At 10-fold higher concentrations (1:100 drug:media), brinzolamide, timolol maleate, and latanoprost actually showed increases in *LOXL1* expression relative to controls. This high-concentration latanoprost treatment (1:100) demonstrated the most pronounced increase in gene expression, which was 41% higher (p<0.003) than the control group. High-concentration brinzolamide and timolol maleate showed increases of 19% (p<0.05) and 26% (p<0.03) above normal, respectively. P-values were obtained using two-tailed *t*-tests of two-samples with unequal variance. No significant increase in expression was seen with brimonidine tartrate with both 1:100 and 1:1,000 concentrations. Thus, drug treatment results in little change in *LOXL1* expression at physiologic drug concentrations (1:1,000) and increased expression at 10-fold higher concentrations. This suggests that the decreased expression levels seen in XFG are not due to drug therapy.

## Discussion

LOXL1 has been shown to be strongly associated with XFS and XFG in every population cohort studied to date. However, the functional significance of the disease associated SNPs is unclear since some cohorts have shown inverse relationships between the initially reported risk alleles. The aim of this study is to see if there is a difference in *LOXL1* expression between normal, XFS, and XFG tissues. We choose lens capsule specimens for two reasons: 1) this tissue is a major site of production of XFS particles and 2) these specimens are easily obtained at the time of surgery and essentially generate in vivo expression data. Our results demonstrate that *LOXL1* gene expression is reduced in lens capsule specimens from XFG but not XFS individuals. The cell culture experiments suggest that this decrease in expression is not due to a medication effect since drug incubation does not lead to a decrease in *LOXL1* expression. Even when the drug concentration was increased ten fold, some of the medications (brinzolamide, timolol, and latanoprost) resulted in an increase (rather than decrease) in *LOXL1* expression. This observation establishes that IOP-lowering medications are unlikely to contribute to the pathological decrease in *LOXL1* expression seen in XFG. Moreover, *LOXL1* expression has been suggested to decrease with age [36] but in our samples, the ages were similar between the three groups with the XFG group having the lowest average age. Therefore, neither age nor medication use explains the differences we observed.

Our results are similar to those reported by Schlotzer-Schrehardt et al. [34] in cadaveric ciliary body specimens in that decreased expression was identified in XFG specimens. They also reported slightly increased expression in early XFS and decreased expression in late XFS. However, we do not have a standard method of distinguishing early versus late XFS and therefore we choose to analyze all the XFS individuals together. Furthermore, we did not genotype our specimens because surgically obtained lens capsules yield very small quantities of mRNA and DNA which limit the analyses that could be performed. However, prior published studies (including that from our patient population) demonstrate a high prevalence of rs3825942 (94%) and rs1048661 (79%) among affected individuals [14]. Therefore, the majority of our specimens likely contain the disease associated SNPs.

Furthermore, *LOXL1* polymorphisms generally have an equal prevalence between both XFS and XFG cohorts. However, in our study, we found decreased expression only among the XFG group and relatively similar expression levels between cataract controls and XFS individuals. The reason for the difference between XFS and XFG is unclear at present and suggests that there may be other modifying genetic or environmental factors that play a role in glaucoma development.

The results of this study suggest a causative functional relationship between *LOXL1* expression and pseudoexfoliation glaucoma. We surmise that decreased *LOXL1* expression promotes the accumulation of pseudoexfoliation particles that appear on histology to clog the trabecular meshwork, collapse Schlemm’s Canal, and lead to increased IOP as seen in XFG [3,37]. If a causative functional relationship can be validated, modification of *LOXL1* expression in affected tissues may represent a novel treatment strategy for this disorder.
